# Development and application of a 6.5 million feature Affymetrix Genechip® for massively parallel discovery of single position polymorphisms in lettuce (*Lactuca spp.*)

**DOI:** 10.1186/1471-2164-13-185

**Published:** 2012-05-14

**Authors:** Kevin Stoffel, Alexander Kozik, Hamid Ashrafi, Xinping Cui, Xiaoping Tan, Theresa Hill, Sebastian Reyes-Chin-Wo, Maria-Jose Truco, Richard W Michelmore, Allen Van Deynze

**Affiliations:** 1 Seed Biotechnology Center, University of California, Davis, CA, 95616, USA; 2 Genome Center, University of California, Davis, One Shields Ave., Davis, CA, 95616, USA; 3 Department of Plant Sciences, University of California, Davis, CA, 95616, USA; 4 Department of Statistics, University of California, Riverside, CA, 92521, USA; 5 Center for Plant Cell Biology and Institute for Integrative Genome Biology, University of California, Riverside, CA, 92521, USA; 6 Nunhems Netherlands B.V., P.O. Box 4005, 6080, AA, Haelen, The Netherlands; 7 Monsanto, Molecular Breeding Technology, 700 Chesterfield Pkwy W, BB34, Chesterfield, MO, 63017, England

## Abstract

**Background:**

High-resolution genetic maps are needed in many crops to help characterize the genetic diversity that determines agriculturally important traits. Hybridization to microarrays to detect single feature polymorphisms is a powerful technique for marker discovery and genotyping because of its highly parallel nature. However, microarrays designed for gene expression analysis rarely provide sufficient gene coverage for optimal detection of nucleotide polymorphisms, which limits utility in species with low rates of polymorphism such as lettuce (*Lactuca sativa*).

**Results:**

We developed a 6.5 million feature Affymetrix GeneChip® for efficient polymorphism discovery and genotyping, as well as for analysis of gene expression in lettuce. Probes on the microarray were designed from 26,809 unigenes from cultivated lettuce and an additional 8,819 unigenes from four related species (*L. serriola*, *L. saligna*, *L. virosa* and *L. perennis*). Where possible, probes were tiled with a 2 bp stagger, alternating on each DNA strand; providing an average of 187 probes covering approximately 600 bp for each of over 35,000 unigenes; resulting in up to 13 fold redundancy in coverage per nucleotide. We developed protocols for hybridization of genomic DNA to the GeneChip® and refined custom algorithms that utilized coverage from multiple, high quality probes to detect single position polymorphisms in 2 bp sliding windows across each unigene. This allowed us to detect greater than 18,000 polymorphisms between the parental lines of our core mapping population, as well as numerous polymorphisms between cultivated lettuce and wild species in the lettuce genepool. Using marker data from our diversity panel comprised of 52 accessions from the five species listed above, we were able to separate accessions by species using both phylogenetic and principal component analyses. Additionally, we estimated the diversity between different types of cultivated lettuce and distinguished morphological types.

**Conclusion:**

By hybridizing genomic DNA to a custom oligonucleotide array designed for maximum gene coverage, we were able to identify polymorphisms using two approaches for pair-wise comparisons, as well as a highly parallel method that compared all 52 genotypes simultaneously.

## Background

Various types of microarrays have been used extensively for gene expression studies and, more recently, for genotyping and marker discovery [1-5]. Affymetrix microarrays in particular offer a massively-parallel approach to genotyping. The basis of identifying polymorphisms, termed single feature polymorphisms (SFPs), is differential hybridization of template RNA or DNA onto 25 bp oligonucleotide probes on the array due to the presence of single nucleotide polymorphisms (SNPs) or small insertion/deletions (InDels). Using this approach, thousands of genes can be queried and simultaneously analyzed allowing whole genome approaches to mapping genes and quantitative trait loci (QTL) discovery [6], as well as determining linkage disequilibrium (LD) [7] and population structure [8,9]. When arrays represent coding sequences, they can also be used for genotyping closely related species [2].

Although Affymetrix expression arrays can be used for genotyping, their drawback is that all except the most recently produced microarrays have been designed with approximately 11 perfect match probes per unigene giving only limited coverage of each gene. Gresham *et al.*[10] showed that an array designed with 25 bp oligos in a 5 bp overlapping tile format had greater sensitivity (ability to detect true SFPs) in yeast and increased specificity (reduced rate of false positives) in calling SFPs. This overlapping tile design offers technical reproducibility and extensive genome coverage if the number of features on the microarray is sufficient.

Genotyping by microarray hybridization has proven to be challenging in species with complex genomes. Microarrays have been successfully used to detect SFPs in small genomes, for example the 13.5 Mb genome of yeast [10], the 145 Mb genome of Arabidopsis [1,8] and, more recently, the 389 Mb genome of rice [3]. Although different algorithms were used for each of these three species, 87% of the SFPs in yeast [10] and 75% of those in rice and *Arabidopsis*[1,3] were independently validated. To identify polymorphisms in the barley genome, complexity was circumvented by using RNA to hybridize to the microarray and 67% [4] to 80% [11] validation of SFPs was achieved. When DNA from barley (5,200 Mb genome) was hybridized directly to the Barley1 GeneChip® a significant overlap between SFPs identified using genomic DNA and those identified and validated using RNA was reported [4]. The increased efficiency reported by Rostoks *et al.* from using RNA is, however, complicated by incomplete and variable transcriptome representation due to tissue- and environment-specific gene expression and false SFP discovery due to alternative splicing or adenylation [4] associated with sampling RNA versus DNA.

Several types of analyses have been implemented for SFP detection from microarray data. Generally, the data have been processed using expression analysis software to correct for background signal variation using Robust Multi-array Analysis (RMA) [12] followed by correction for overall signal variation by quantile normalization across arrays [13]. To call SFPs, a modified T-test [1], Robustified Projection Pursuit (RPP) [11] and SFP de-viation [5] have been developed to first estimate the normalized hybridization of a reference set of probes and then test with appropriate statistics or ratios for differential hybridization of specific probes across genotypes. In addition, a maximum likelihood procedure using the source of sequence on the chip as a reference was deve-loped by Gresham *et al.*[10] to take advantage of overlapping tile data to call SFPs. As each microarray and experiment design tends to be different, new methods for analysis have been developed in attempts to gain greater specificity and sensitivity.

Cultivated lettuce, *L. sativa*, has substantial genetic and genomic resources including approximately 76,000 ESTs and another 20,000 to 50,000 ESTs in each of four related species (http://compgenomics.ucdavis.edu/). Furthermore, several mapping populations have been developed including a reference mapping population of 214F_7_ recombinant inbred lines (RILs) between *L. sativa* cv. Salinas and *L. serriola* acc. US96UC23 ([14]; RW Michelmore *et al*., unpublished). This population segregates for multiple agronomic, disease resistance and quality traits. It has approximately 1,500 mapped DNA markers comprised of approximately 700 mapped unigenes with the remainder amplified fragment length polymorphisms (AFLPs), restriction fragment length polymorphisms (RFLPs) and simple sequence repeats (SSRs) [14]. The large number of available sequences and recombinant inbred lines provide ideal resources for further marker discovery and high density mapping. Considerable genetic resources are also available within germplasm collections of *L. sativa*. Furthermore, several *Lactuca* species have variable cross-compatibility with *L. sativa*[15] and represent a diverse genetic resource for investigations of novel alleles and population structure.

In this paper, we describe the development of a microarray designed to provide extensive gene coverage and maximize detection of SFPs for marker discovery and genotyping in lettuce. We analyzed the parents of the reference *L. sativa* x *L. serriola* mapping population to demonstrate that DNA representing complex genomes (2,639 Mb) [16] can be effectively hybridized onto microarrays. Parameters affecting DNA hybridization and accurate detection of polymorphism were optimized. Algorithms from West *et al.*[5] and Borevitz *et al.*[1] were modified to take advantage of the overlapping tile design to detect polymorphisms. The use of the multiple probes covering a single position led to the identification of single position polymorphisms (SPPs). Additionally, we assessed SPPs in a diverse panel of *Lactuca* species concentrating on the cultivated *L. sativa.*

## Results

### Identification of a non-redundant consolidated unigene set from *Lactuca spp.* for design of an oligonucleotide array

A consolidated *Lactuca* unigene set (CLUS) was created using stringent CAP3 conditions [17]. This set represented all the currently available genes in March 2006 that had been identified in cDNA libraries prepared from *L. sativa* cv. Salinas, plus additional genes that were not present in those libraries from four other related species of *Lactuca* (see Methods). The selection of unigenes was performed reiteratively in order of increasing genetic divergence from *L. sativa*; first, unigenes from *L. serriola,* US96UC23, were analyzed by TBLASTX followed by unigenes from *L. saligna*, *L. virosa* and lastly *L. perennis*. The final set comprised of 26,809 unigenes from *L. sativa plus* 4,065*,* 1,391, 1,686 and 1,686 from *L. serriola, L. saligna*, *L. virosa*, and *L. perennis* respectively, totaling 8,828 unigenes from the four other *Lactuca* species (Table 1). This resulted in a final CLUS of 35,637 unigenes (Table 1). An additional 151 unique *L. sativa* genomic sequences possessing a TBLASTX hit (<e-10) to the *Arabidopsis* genome and characterized mRNA sequences were then mined from Genbank and added, resulting in a final list of 35,788 *Lactuca* sequences that were submitted to Affymetrix for probe design.

**Table 1:** The number of ESTs and Unigenes for each species before and after filtering

	***L. sativa***	***L. serriola***	***L. saligna***	***L. perennis***	***L. virosa***	**Total**
ESTs	76043	52034	28851	28066	28335	
Unigenes	29417	22327	11990	12661	12733	
Unigenes after filtering	26809	4065	1391	1686	1686	35637

### Design of microarray with overlapping probes

In collaboration with Affymetrix, probes from both sense and anti-sense strands were selected to create a 2 bp overlapping tiling path (See Additional file 1: Figure S1). The resulting 11.4 million candidate probes designed from the 35,788 submitted sequences were triaged down to ~6.5 million that could be accommodated on the chip through a series of steps based on: 1) Affymetrix probe quality score (> 0.25) except for a select set of unigenes with putative polymorphisms where probes with a quality score above 0.1 were retained. 2) probes matching mitochondrial or chloroplast genomes were discarded. 3) Probes that matched to more than one target were synthesized only once on the chip and computationally associated to corresponding unigene for analysis.

In addition to lettuce probes, background and standard Affymetrix control probes [18] were included on the microarray. In order to determine background hybridization signal, 13,567 anti-genomic (AG) background probes were synthesized on the microarray, with an average of 1,355 probes representing each G/C bin (probes with the same number of guanines and/or cytosines in the 25 bp probe). These AG probes represent sequences that had no BLAST hits in GenBank at the time of chip design. The use of AG probes reduced the number of probes included on the chip for background correction by 99% compared to the use of mismatch probes (allocated to half of the array positions in pre-vious designs), without substantially compromising the ability to perform accurate background correction [19]. From 8,000 visually interrogated EST contigs, ~2,000 putatively polymorphic regions (50 to 100 bp) were represented from 1,184 contigs.

In total, 6,410,923 lettuce probes representing 35,788 unigenes were synthesized on the microarray. The average and median number of probes representing a unigene were both 187, with ~80% of the unigenes being represented by 50 to 275 probes per sequence (See Additional file 2: Figure S2). Each unigene had an average of 591 bps and median of 603 bps covered by probes; the average number of contiguous stretches of overlapping probes per unigene is 3.1. Due to the selection parameters described above, a contiguous overlapping tile across the unigenes was not possible. Consequently, the average and median lengths of the contiguous stretches of overlapping probes within probe sets are 190 and 120 bps, respectively. Regions of high or low G/C content were sparsely covered by probes (See Additional file 3: Figure S3) likely due to the removal of probes with low Affymetrix probe quality scores. The total number of probes on the array was 6,482,479.

### Preparation and hybridization of genomic DNA to the lettuce GeneChip

**Figure 1:**
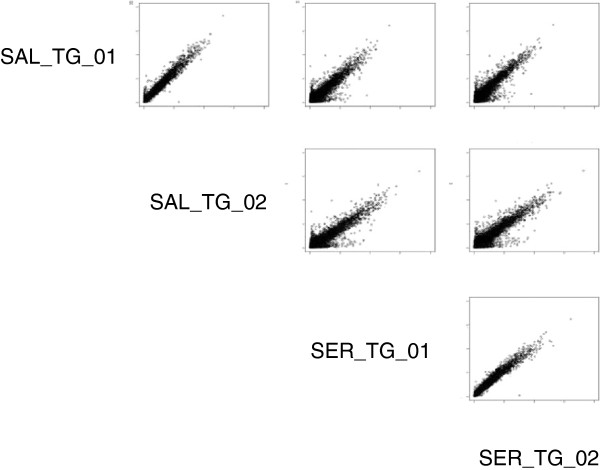
**Pair-wise scatter plots of 600,000 probes from SAL and SER.** Pair-wise scatter plots of RMA background corrected hybridization values for 600,000 random probes for two technical replicates of *L. sativa* cv. Salinas (SAL_TG_01, SAL_TG_02) and *L. serriola* acc. US96UC23 (SER_TG_01, SER_TG_02). Comparisons across species show larger deviations than those between replicates

Large amounts of high quality genomic DNA were a critical prerequisite for robust hybridization signals (see below). To meet these criteria we compared fragmented genomic DNA to amplified DNA using whole genome amplification (WGA) from Sigma (see Methods). Analysis of scatter plots comparing hybridization intensities resulting from amplified and unamplified genomic DNA revealed that WGA resulted in marked biases (See Additional file 4: Figure S4). High quality DNA was extracted for each of *L. sativa* cv. Salinas and *L. serriola* acc. US96UC23. Each of these extractions were hybridized twice providing two technical replicates and hybridization intensities were evaluated using scatter plots of 600,000 random probes (Figure 1) or probes belonging to a set of ultra-conserved sequences (http://compgenomics.ucdavis.edu/compositae_reference.php). Comparison between replicates showed a nearly 97% correlation while between species showed approximately 93% correlation indicating infrequent hybridization differences as expected with low rates of polymorphism between the two species.

**Figure 2:**
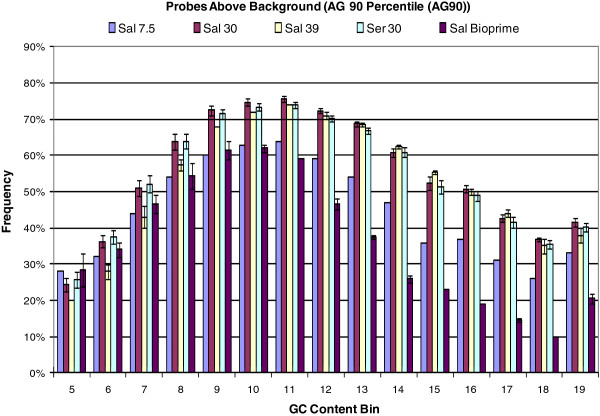
**Frequency of probes with hybridization signals greater than background per GC bin.** Frequency (y-axis) of lettuce probes on the GeneChip®, per GC content bin (x-axis), with a hybridization signal that is higher than the 90 percentile of the hybridization signal of the anti-genomic probes with corresponding GC content. Results are shown for *L. sativa* cv. Salinas hybridizations with 7.5, 30 or 39 μg of gDNA, *L. serriola* with 30 ug gDNA, *L. sativa* cv. Salinas hybridizations with Genomic DNA fragmented and labeled using BioPrime (Invitrogen, Carlsbad, CA, USA), and *L. serriola* acc. UC96US23 hybridizations with 30 μg of gDNA

We investigated several methods for fragmentation and labeling of genomic DNA including the Bioprime kit (Invitrogen, Carlsbad, CA, USA). Although incorpo-ration of biotinylated dCTP during amplification of target sequence via random priming resulted in elevated hybridization intensities compared to end-labeling of fragmented DNA, hybridization intensity of both lettuce and background probes increased with GC content dramatically (See Additional file 5: Figure S5), resulting in a decreased number of informative probes (probes above background) with high GC content (Figure 2). We concluded that direct fragmentation of genomic DNA by digestion with DNase I and end-labeling was the most cost and time effective, least biased (due to the lack of amplification and selection steps), and most informative method for preparing genomic DNA for hybridization to microarrays.

**Figure 3:**
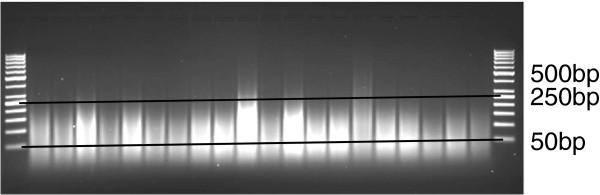
**Agarose gel electrophoresis of genomic DNA extracted from lettuce.** Two μls from a 30 μg fragmentation of lettuce genomic DNA with DNase I was separated on 2% agarose gel and visualized by ethidium bromide staining. Lengths in bp the O’GeneRuler™ 50 bp DNA ladder (Fermentas, Glen Burnie, MD, USA) are shown. Samples were accepted for labeling provided that the majority of fragments were within 50 to 250 bp

To determine the quantity of genomic DNA required to achieve adequate hybridization, three different quantities of genomic DNA, 7.5 (the amount typically used in cDNA hybridizations), 30, and 39 μg, from *L. sativa* cv. Salinas were sheared with DNase I and end-labeled. The number of lettuce-specific probes with fluorescent intensities above the 90^th^ percentile intensity for the AG control probes in the same GC bin was determined for each sample (e.g. Figure 2). The 30 μg sample of fragmented DNA yielded the highest percent of probes above the 90^th^ percentile (62%) in these GC bins when compared to the 7.5 (45%) and 39 μg (60%) DNA samples (Figure 2). DNase I fragmentation conditions were consequently optimized for this amount to consistently provide fragments predominantly between 50 and 250 bp in length (Figure 3). Thirty micrograms was selected as the standard concentration of genomic DNA for subsequent experiments.

### Improvement of the algorithm for detecting polymorphisms

**Figure 4:**
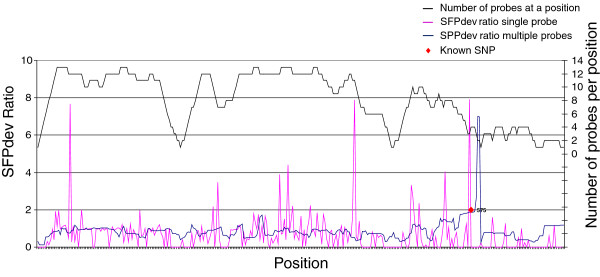
**Graphical representation of SFP vs. SPP calls along a contig.** The x-axis shows the position of a probe along a contig. The y-axis shows the difference in the average weighted SFPdev or SPPdev values between the two genotypes. The SPP analysis detected only the true SNPs while the SFP analysis indicated multiple false positives

**Figure 5:**
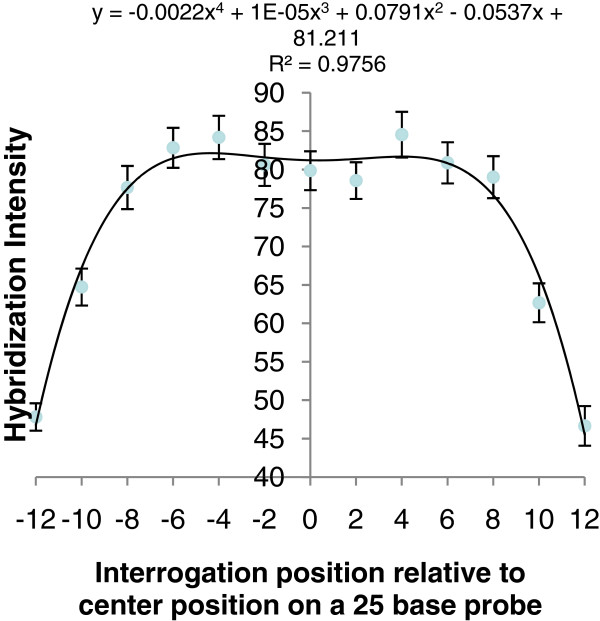
**A graphical representation of the equation used to determine a weighting factor at each position.** The graph shows the mean hybridization difference between *L. sativa* cv. Salinas and *L. serriola* acc. US96UC23 (y-axis) plotted against the 2 bp interrogation position relative to the central position of the probe being measured (x-axis). The equation above was used for calculating the weighting factor used in the two-genotype comparison algorithms

The algorithms developed previously by West *et al*. [5], were modified to take advantage of the tiling design of the lettuce GeneChip®. The new algorithm calculated the SFPdev value for each of the probes that overlap a given position and then performed a sliding window analysis to calculate an average weighted SFPdev value for each 2 bp position across the unigene covered by at least one probe. This strategy markedly reduced background noise relative to individual probe measurements (Figure 4). Additionally, removal of probes below the 90^th^ percentile of AG probes in the same GC bin increased confidence in calls while identifying polymorphisms missed by inclusion of poorly performing probes (See Additional file 6: Figure S6). An empirically determined weighting factor based on sensitivity of bases within an oligo to sequence polymorphisms (Figure 5) was included in our custom algorithm to give more significance to the 16 most centrally located bases in a probe. This weighting factor allowed us to retain probes nearest the edges of tiling blocks where polymorphism could be found, but reduced the emphasis given to SPPs detected by those probes rather than disregarding them completely, allowing users to potentially filter out or retain them. As our algorithm uses data from multiple informative features that interrogated each 2 base pair position rather than a single feature, we designated the polymorphisms detected as single position polymorphisms (SPPs).

We also modified the algorithm described by Borevitz *et al.*[1] for detection of SFPs to exploit the tiling array design. This modified SFP algorithm (MSA) interrogated all weighted probes above background at every 2 bp position, and calculated a *D-stat* similar to that described by Borevitz *et al.*[1]. This, however, was done for each position rather than each probe. A false disco-very rate was calculated for each threshold cutoff value for both pairwise analyses using permutation analysis as described by Borevitz *et al.*[1].

### **Detection of SPPs between*L. sativa cv. Salinas*and*L. serriola acc***. **US96UC23**

Genomic DNA from *L. sativa* cv. Salinas (SAL) and wild *L. serriola* acc. US96UC23 (SER) were hybridized to the GeneChip® in three technical replicates. The Affymetrix .CEL file data were background-corrected by RMA and quantile-normalized across all chips. The data were analyzed for SPPs using both our modified SFPdev and MSA algorithms. SPPs were filtered to require a minimum 4 bp range and two informative probes covering the interrogated position to increase confidence in the SPPs called. SPPs were defined by the range of positions (bp) that met a selected FDR value of 0.1 and a Delta value of 0.2 for the SFPdev and MSA methods, respectively. Furthermore, only the SFPdev values with a ratio (SAL/ SER) less than 1 were considered, as values above one had an actual FDR of 79%. With these requirements the SFPdev method identified 40,462 SPPs in 19,345 contigs; while 40,960 SPPs in 18,290 contigs were identified with the MSA method. The coincidence of reported SPPs between the two methods showed that 73.6% of SPPs detected by the SFPdev method were found by MSA, and 81.1% of SPPs reported by MSA were found by SFPdev.

To provide an independent assessment of polymorphisms, Illumina mRNA-seq reads (IGA set) from *L. sativa* cv. Salinas and *L. serriola* acc. US96UC23 were aligned to the unigenes used for chip design to identify a set of SNPs. Identified SPPs were compared to this set as well as SPPs identified and mapped in the reference RIL population [20] to validate the true and false positive rate of our detection methods. Further, if the SPP regions were identified as being duplicated in the genome they were removed as they were falsely called one third of the time (Methods). Using the modified SFPdev method with an estimated FDR of 0.1, 23,835 SPPs with an actual FDR of 24.35% were identified. Our MSA method at a delta value of 0.2 with an estimated FDR of 36.45% filtered for the same conditions as above called 23,075 SPPs with an actual FDR of 14.46%. Table 2 shows the relative numbers of SPPs identified and their corresponding FDRs for each method.

**Table 2:** Observed FDR for each cut-off value for the three SPP prediction methods

			
**a.**	**MSA**	**Observed**	**Permuted**
	Delta 0.2	14.50%	36.45%
	Delta 0.4	13.80%	17.54%
	Delta 0.6	11.00%	10.85%
	Delta 0.8	10.20%	8.34%
	Delta 1.0	9.80%	7.27%
	Delta 1.2	9.10%	6.80%
	Delta 1.4	8.50%	6.58%
	Delta 1.6	7.90%	6.45%
**b.**	**SFPdev**	**Observed**	**Permuted**
	FDR 0.10	24.30%	10.00%
	FDR 0.05	21.40%	5.00%
	FDR 0.01	20.10%	1.00%
**c.**	**DP Analysis**	**Observed**	**Permuted**
	SFPDev 1.2	2.80%	N/A
	SFPDev 1.5	1.70%	N/A
	SFPDev 2.0	1.10%	N/A

Table 2 shows the observed and permuted FDRs for each of the three SPP detection methods. **a)** Delta values from 0.2 to 1.6 for MSA analysis show decreasing FDRs as stringency increases. At values lower than 0.6 the observed values surpass those expected by permutation analysis. **b)** Three cutoff values for SFPdev were reported from 10% to 1%. Observed values did not change drastically as permuted stringencies increased. **c)** Observed FDR values in DP analysis were low and did not drastically change as cutoff values increased.

**Figure 6:**
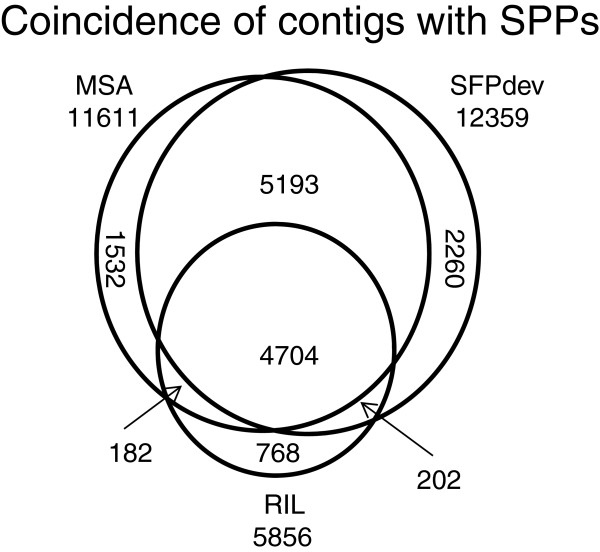
**Venn diagram showing the overlap of contigs containing SPPs among the three SPP identification methods.** A Venn diagram of the three SPP identification methods shows the overlap of contigs containing SPPs between each method. The two pair-wise comparison methods had the largest overlap of identified contigs. MSA – pairwise comparison using a modified version of the algorithm described by Borevitz *et al.*[1]. SFPdev – pairwise comparison using a modified version of the algorithm described by West *et al.*[5]. RIL - massively parallel approach looking at the distribution of SPP calls for all individuals in a diversity panel

The modified SFPdev and MSA methods provide pair-wise comparisons but are too computationally intensive to assess polymorphisms between many pairs of lines. Therefore, a third analysis was performed using the RIL algorithm method described by Truco *et al*. [20]. All 2 bp windows were assessed for bimodal distributions in hybridization values from a diverse panel of genotypes composed of 52 accessions from five *Lactuca* species including the parents, Salinas and US96UC23, of the reference RIL mapping population. Selection of SPPs that were identified as polymorphic between these two parental lines using this method identified 18,237 SPPs. This set was filtered with the same criteria as described above and included no inconsistent data points yielding an actual FDR of only 2.83%. A Venn diagram of contigs with SPPs was created to show the coincidence of potentially polymorphic contigs detected using each method (Figure 6). We included polymorphic contigs rather than SNPs detected in this figure as the SPPs reported are ranges. These ranges often covered more than one SNP rendering it impossible to determine which polymorphism was the contributor to the detected SPP. Overall, the majority of contigs containing SPPs identified by each of the three methods coincide (4,707). The two methods that have the largest overlap (9,897) were the two pair-wise comparison methods (MSA and SFPdev).

### Analysis of a Diversity Panel (DP)

The massively parallel genotyping of 52 lines (41 *L. sativa*, 5 *L. serriola,* 3 *L. saligna,* 2 *L. virosa,* and 1 *L. perennis,* Table 3) using the modified RIL algorithm was employed to investigate inter- and intra-specific polymorphisms. The SPPs were filtered to allow zero missing and zero inconsistent data, coverage by a minimum of two probes at the interrogated position, a minimum SPP width of 4 bp and a SFPdev ratio of 1.2. This resulted in 74,034 SPPs from 23,144 contigs. The SPPs detected in the diversity panel, were further filtered to exclude any SPPs with a duplicate pattern of markers across genotypes within the same unigene yielding 46,237 distinct haplotypes. We were able to identify 43,464 SPPs that showed polymorphism between species but not within the five species in the diversity panel. Most were private to *L. perennis*, followed by *L. virosa* and *L. saligna*.

**Table 3:** Individuals in the diversity panel and their relative species and horticultural class within *L. sativa*

**Panel ID**	**Species**	**Horticultural class**	**Panel ID**	**Species**	**Horticultural class**
BSP001	*L. sativa*	Butterhead	SAL	*L. sativa*	Iceberg
BSP002	*L. sativa*	Butterhead	UCD004	*L. sativa*	Iceberg
BSP003	*L. sativa*	Butterhead	UCD006	*L. sativa*	Iceberg
BSP004	*L. sativa*	Butterhead	BSP021	*L. sativa*	Leafy
BSP005	*L. sativa*	Butterhead	BSP022	*L. sativa*	Leafy
DIANA	*L. sativa*	Butterhead	BSP023	*L. sativa*	Leafy
OLOF	*L. sativa*	Butterhead	BSP024	*L. sativa*	Leafy
UCD005	*L. sativa*	Butterhead	BSP025	*L. sativa*	Leafy
UCD007	*L. sativa*	Butterhead	UCD003	*L. sativa*	Leafy
BSP006	*L. sativa*	Cos	BSP026	*L. sativa*	Leafy
BSP007	*L. sativa*	Cos	BSP027	*L. sativa*	Leafy
BSP008	*L. sativa*	Cos	BSP028	*L. sativa*	Leafy
BSP009	*L. sativa*	Cos	BSP029	*L. sativa*	Leafy
BSP010	*L. sativa*	Cos	BSP030	*L. sativa*	Leafy
UCD002	*L. sativa*	Cos	UCD014	*L. sativa*	Oil
BSP011	*L. sativa*	Batavia	SER	*L. serriola*	
BSP012	*L. sativa*	Batavia	UCD010	*L. serriola*	
BSP013	*L. sativa*	Batavia	UCD011	*L. serriola*	
BSP014	*L. sativa*	Batavia	UCD012	*L. serriola*	
BSP015	*L. sativa*	Batavia	UCD013	*L. serriola*	
BSP016	*L. sativa*	Batavia	LSALIGNA	*L. saligna*	
BSP017	*L. sativa*	Iceberg	UCD01	*L. saligna*	
BSP018	*L. sativa*	Iceberg	UCD017	*L. saligna*	
BSP019	*L. sativa*	Iceberg	UCD018	*L. virosa*	
BSP020	*L. sativa*	Iceberg	UCD019	*L. virosa*	
GREENLAKE	*L. sativa*	Iceberg	UCD020	*L. perennis*	

Note : Batavia and Iceberg are sub-classes of the Crisphead class.

Polymorphism within *L. sativa* is most pertinent to breeding efforts. To survey polymorphism within this species, SPPs were filtered from the 74,034 previously described to include only those polymorphic within *L. sativa* resulting in 8,211 SPPs on 4,412 contigs. The leafy and Batavia crisphead plant types had the most diversity while iceberg crisphead contained the least (Table 4). However, 2,343 SPPs were identified even within the iceberg type allowing distinction of cultivars within this genetically narrow plant type. Several SPPs showed diversity within one plant type, while being monomorphic in other *L. sativa* types.

**Table 4:** Number of SPPs and Contigs containing SPPs identified for each class/species by DP analysis

			
**a)**	**Species**	**Contigs**	**SPPs**
	*L. sativa*	394	507
	*L. serriola*	293	431
	*L. saligna*	3,651	6,729
	*L. virosa*	3,315	7,023
	*L. perennis*	13,305	28,774
	Total Markers	20,958	43,464
**b)**	***L. Sativa* types**	**4,412**	**8,211**
	Butterhead	1,956	3,531
	Cos	1,931	3,420
	Batavia	2,135	3,840
	Iceberg	1,279	2,343
	Leafy	3,467	6,178
	Oil only	364	557

a) The number of contigs containing SPPs and the number of SPPs private to the genotypes within a species. These SPPs distinguish all the genotypes in a species from all others in the panel. b) The number of contigs containing SPPs and the number of SPPs for each *L. sativa* horticultural class. These markers are polymorphic within a class but not specific to that class.

**Figure 7:**
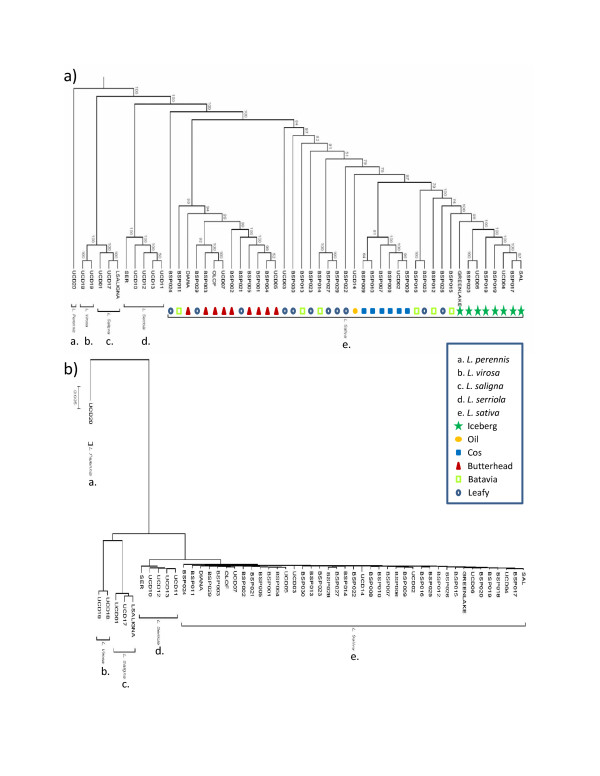
**Phylogenetic trees estimating the relationship of all individuals in the diversity panel. a)** Dendogram estimating the relationship of genotypes in the diversity panel. Bootstrap values indicate the confidence in branch positioning. **b)** Representative phylogram showing the relative relatedness of individuals. Each species is monophyletic

A phylogenetic analysis was then performed with the PHYLIP 3.69 package [21]. Using the filtered 46,237 marker set for the 52 genotypes, a bootstrap consensus tree was constructed (Figure 7a). A representative phylogram (Figure 7b) estimates the genetic differences between the genotypes in the panel. The *L. sativa* genotypes separated into two distinct clades with 100% bootstrap value separating butterheads from the cos and majority of crisphead types. The leafy lettuce genotypes showed high variability locating within both the butterhead and cos/crisphead clades. Iceberg varieties showed the least amount of polymorphism and grouped together in a monophyletic clade with 100% bootstrap support. One genotype, BSP024, showed a *L. sativa*-like morphology but has a seed shattering phenotype characteristic of the wild species, positioned between the wild species and the remaining *L. sativa* with 100% support. Upon further analysis, branch lengths of *L. sativa* genotypes indicate similar divergence from their common ancestor with the exception of BSP024 and UCD14 (oil type) (See Additional file 7: Figure S7).

**Figure 8:**
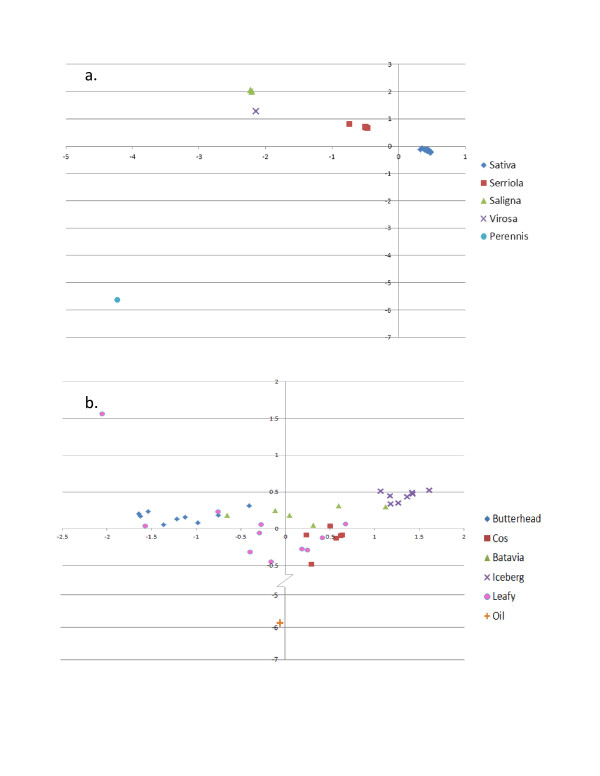
**Two dimensional scatter graphs of eigenvalues from principal component analysis.** The first two significant eigenvalues from principal component analyses performed with SAS software PRINCOMP procedure are plotted against each other to show resolution within species or classes. **a)** Eigenvalues significant at P < 0.0001 from principal components one and two are plotted against each other and show clear resolution of species. **b)** Eigenvalues one and three were both significant at P < 0.0001 and were plotted against each other. The y-axis was altered to show clearer resolution in non-oil genotypes. Batavia and Leafy classes show distribution through the scatter plot similar to that seen in Figure 7a and 7b

Two separate principal component analyses (PCA) were performed on the entire diversity panel as well as on just *L. sativa.* The first three eigenvalues account for 71.4% of the variation seen across all five species. While each of the three principal components (PC) were significant at <0.0001 when separated by species, two dimensional scatter plots of PC values for each genotype using the first two PC show clear separation of species (Figure 8a). When considering separation between types within *L. sativa*, the first three PCs accounted for 27.6% of the variation within the population with the first and third PCs being significant at <0.0001. A two dimensional plot (Figure 8b) of the first and third PC values showed some overlap of types consistent with Figure 7a. Two genotypes, UCD14 and BSP024, are again outliers and show drastic deviation from the rest of the *L. sativa*s (See Additional file 7: Figure S7 and Figure 8b).

## Discussion

Highly parallel genotyping has become an important component of genomics. Hybridization of genomic DNA and RNA to microarrays has been used in the past for detection of polymorphisms between genotypes [1,4,5,10,22]. However, the previously available arrays for complex genomes only provided limited transcriptome coverage. We developed an array designed to maximize transcriptome coverage while maintaining the possibility of performing other analyses. Our custom designed Lettuce GeneChip® combined the benefits of overlapping probes across unigenes, similar to that demonstrated by Gresham *et al.*[10] for yeast, with the use of anti-genomic probes to maximize the possible coverage of unigenes while maintaining the sensitivity to detect polymorphisms and retaining appropriate controls to normalize and correct for background noise. The tiling path design allows for multiple assessments of hybridization differences between lines for single positions rather than single assessments of a few positions as obtained with most expression arrays. We developed custom scripts for analysis of our hybridization data taking into account the multiple probes covering a single position as well as filtering out poorly performing probes. We used recent advances in high throughput sequencing technology to validate our SPP calls as well as filter out potentially unreliable data.

Genomic DNA and cDNA are two options for hybridization to an array for SFP detection. The decision of which to use becomes more difficult as genome size and complexity increases. DNA as well as cDNA are both viable targets for species with smaller genomes such as Arabidopsis [1,5] and rice [3,23]. However, with larger and more complex genomes such as barley, cDNA was indicated as a more reliable option for hybridization even with the added difficulty of subtracting out expression effects [4]. The genome of lettuce is nearly 17x larger than Arabidopsis although it is half the size of barley. Given the difficulty of accounting for spatio-temporal expression effects as seen in cDNA, we focused on developing methods to use genomic DNA. Rostoks *et al.*[4] suggested that genomic DNA may be a feasible target in larger genomes with added replication. With the redundancy of the overlapping probes in the lettuce array, the need for additional replication was reduced because they provide technical replicates within a chip. The need for additional replication may also have been reduced by using elevated amounts of genomic DNA and the use of end-labeling rather than BioPrime may have increased the reliability of calls. The protocol used for hybridization of lettuce genomic DNA was also subsequently highly effective for pepper (genome size = 3,000 MB) and other Solanaceae [24]. Furthermore, the use of genomic DNA is a desirable target because SFPs identified using cDNA may be a result of alternate splicing or gene expression differences [25]. Rostoks *et al*. [4] indicated that 40% of the SFPs they identified may have been falsely called and partially explained them as being mRNA structural variants. They also reported a high predicted false positive rate of 22% (their mid-stringency cutoff value) for SFPs detected using genomic DNA. We concluded that fragmented, end-labeled genomic DNA provided a suitable target for detection of polymorphisms while reducing false positive sequence polymorphism (2 to 24% in our experiments, Table 2).

The overlapping tile design increases the likelihood of detecting polymorphisms due to redundancy at individual positions, coverage along the contigs and optimal position of the SNP within a probe [1,11]. Furthermore, the number of probes and hence the possible genome coverage was increased by substituting mismatch probes with AG probes for background correction and normalization of data. Because the peripheral 1 to 6 bases of a 25 bp oligonucleotide are less sensitive than the central bases, in terms of detecting sequence polymorphisms [1,4], Figure 4, [11], the tiling strategy reduces the loss of coverage due to probe position. The number and reliability of SPP calls in our experiments demonstrates that the overlapping tiling array design has improved coverage, sensitivity and specificity to detect polymorphisms.

SPP calls were validated using several approaches. The data from the two pair-wise comparisons (MSA and SFPdev) yielded 20 to 41 thousand and 27 to 40 thousand SPPs respectively, depending on the criteria used for specificity and sensitivity. When SPPs from MSA and SFPdev were compared to the 51,552 SNPs detected between RNAseq reads of Salinas and US96UC23, 61.5% and 57.8% were found in or within at least 8 bp of the SPP range respectively, similar to that described by Gresham *et al*. [10]. However, because of the high FDR associated with duplicated sequences, SPPs that were found to have a duplicated locus within the chip assembly, the gene space assembly or the genome assembly were removed from consideration; one third of the SPPs called that had duplicated loci did not contain a SNP in any of our validation tests. These identified SPPs likely were due to differences between paralogs rather than alleles at a single locus. Due to the increased redundancy (up to 107 genetic replicates) provided by the mapping population of 213 RILs compared to the pair-wise comparison of the parents, SPPs in the SFPdev and MSA pair-wise comparisons that coincided with SPP mapped by Truco *et al*. [20] but were absent of a SNP were considered real. Removal of duplicated loci and inclusion of mapped SPPs provided a balance between false positive and false negative rates and allowed us to optimize FDR while still discovering a high number of SPPs. Taking into consideration the lower observed FDR (Table 2) we concluded that the MSA method performed best as a pairwise comparison; however using multiple detection methods would yield a higher confidence in the subset of SPPs identified by both methods.

The SPPs identified in the diversity panel (DP) that were polymorphic between *L. sativa* cv. Salinas (SAL) and *L. serriola* acc. US96UC23 (SER) showed a low FDR. However, as a result of the filtering, sensitivity of this analysis (detection of true positives) was reduced compared to the two-genotype analyses by MSA and SFPdev. Specific analysis of the DP data for regions containing known SNPs showed that SFPdev values would have been significant in a pair-wise comparison, between SAL and SER but due to inclusion of data from all genotypes in the DP, the two were not called as polymorphic (data not shown). The lack of some called SPPs in the DP may be due to larger genetic differences between *L. perennis, L. virosa,* or *L. saligna* relative to *L. serriola* and *L. sativa.* As a result of smaller hybridization differences between the more closely related genotypes, genotypes differing at a locus may have been grouped together reducing the number of SPPs called between the two genotypes. Consequently, the DP analysis showed a lower false positive rate, but a higher false negative rate when comparing SAL and SER to sequence and mapping data.

As part of our goal was to investigate the diversity and relationships of the genotypes in the DP, SPPs identified by the DP analysis were evaluated. Removal of SPPs in duplicated regions with inconsistent data or missing data (see Methods) was a reasonable method of removing unreliable data as these data may be from poorly performing probes in one or all replicates, heterozygous loci, paralogous genes or deleted genes. There was not a large difference in the observed FDRs for the three SPFdev cutoff values (1.2, 1.5, & 2.0) for the DP analysis; so in order to maximize the number of markers used in our phylogenetic analysis and principal component analysis, we used the least stringent cutoff value of 1.2. As the assumptions for analysis with the PHYLIP [21] package were not violated with the large number of markers, they were left as independent. To meet the constraints of the PC analysis software, markers were limited to those that were mapped.

The markers discovered in our DP analysis were used to generate a phylogenetic tree showing species separation with 100% boot strap support. *L. virosa* and *L. saligna* are sexually incompatible species with *L. sativa*[26] and appear to be more closely related to each other than to other species in the DP. Our data supports the conclusion by Kesseli *et al.*[27], that these two species are not progenitors of *L. sativa*. By limiting markers to those polymorphic within cultivated lettuce we are able to separate most genotypes into classes representing each of the plant types. The butterhead type formed a distinct clade from the iceberg and cos types with 100% bootstrap support. However, the leafy type and the Batavia type both showed a wide distribution across the *L. sativa* clade. This is not unexpected and may reflect admixture between types during breeding programs. Alternatively, this distribution may indicate that these types are artificial polyphyletic groups based on loose morphological criteria [28]. The leafy types are non-heading with a broad range of leaf morphology [28,29]. Batavia types vary from heading to non-heading phenotypes. Batavia and iceberg cultivars are both considered crisphead types [28]; however our phylogenetic and PC analyses showed that the two did not cluster together and are significantly different from each other (See Additional file 8: Table S1).

Rapid advancements in sequencing technology today are changing the methods for genetic analyses. Microarray technology presented in this paper yielded an in depth analysis of diversity for lettuce germplasm separating even closely related lines such as the crisphead class. It also has potentially several other uses including: detection of copy number variants, splice site identification, expression analysis or use with other species within the Compositae. The SPPs identified in this study were highly reproducible and showed similar false positive results to current sequencing methods in the literature [30,31]. This technology has also been used to create an ultra-dense, inter-specific genetic map between *L. sativa* cv. Salinas and *L. serriola* acc. US96UC23 to dissect phenotypic traits as well as validate and align genomic assemblies of lettuce into chromosomal linkage groups [20].

## Conclusion

We designed and exploited a custom lettuce microarray using an overlapping tiling path and by using anti-genomic probes rather than mismatch probes to provide comprehensive unigene coverage. Our protocols for genomic DNA preparation and labeling, assisted by positional vs. feature-based analyses reliably identified DNA polymorphisms using both pair-wise genotype comparisons as well as a highly parallel comparison within a diverse panel of genotypes including five species and focused on the cultivated *L. sativa*. The phylogenetic and principal component analyses clearly distinguished species while the analysis of *L. sativa* supports previous analyses of cultivated lettuce and revealed differences among the more heterogeneous horticultural types as well as polymorphisms within the most genetically narrow type.

## Methods

### EST assemblies of *Lactuca spp.*

ESTs for five *Lactuca* species (*L. sativa, L. serriola, L. saligna, L. virosa and L. perennis*) generated as part of the Compositae Genome Project (http://compgenomics.ucdavis.edu) were trimmed to phred scores of 15 and vector sequences were removed with custom Perl scripts (https://code.google.com/p/atgc-xyz/source/browse/#svn%2Ftrunk). Trimmed ESTs for each of the five species of *Lactuca* were assembled separately using CAP3 [17]. To minimize assembly of paralogous sequences, stringent assembly parameters were used for CAP3 (95% identity, 100 nt minimum overlap). The assemblies were combined iteratively using *L. sativa* as a base and adding unique sequences from individual species in order of genetic distance from *L. sativa*.

### DNA extraction

Genomic DNA was extracted following the protocol described by Kozik [32] with minor modifications. Frozen leaf tissue from three-week old, greenhouse-grown plants (2.5 g fresh weight) was ground in liquid nitrogen. Fifteen ml of 2X extraction buffer (100 mM Tris–HCl pH 8.0, 1.4 M NaCl, 20 mM EDTA, 2% w/v CTAB, 10 μl/ml β-mercaptoethanol) was added to the tissue, mixed and incubated at 65°C. One volume of chloroform:isoamyl alcohol (24:1) (ChIA) was added and mixed, and the sample centrifuged at 3,500 rpm for 20 min twice. The aqueous phase was then transferred and 3 to 3.5 volumes of precipitation buffer (50 mM Tris–HCl pH 8.0, 10 mM EDTA, 1% w/v CTAB) were added. The sample was incubated overnight at room temperature to precipitate the DNA and then centrifuged at 3500 rpm for 15 min. The DNA pellet was washed with dH_2_O and centrifuged for 10 min; 5 ml of 1.5 M NaCl and 6 μl of 10 mg/ml RNaseA was added to the pellet and incubated at 37°C until completely re-suspended. A chloroform extraction was performed as above to remove RNaseA and any additional contaminants. The aqueous phase was collected and DNA was precipitated and washed with ethanol. Samples were centrifuged again for 10 min at 3,500 rpm. The pellet was allowed to dry then re-suspended in 100 μl ddH_2_O. The sample was diluted 1:100 and run on a 1% agarose gel with known amounts of uncut lambda DNA to estimate the concentration of the genomic DNA. The 2.5 g of starting material yielded approximately 200 μg of genomic DNA, which was sufficient for hybridization to six chips at 30 μg per chip.

### Whole genome amplification

The GenomePlex® Complete WGA kit (WGA, Sigma-Aldrich Corp, St. Louis, MO, USA) was evaluated for whole genome amplification. The maximum amount of genomic DNA that could be accommodated by the WGA kit (20 ng) was amplified and then re-amplified using the GenomePlex WGA re-amplification kit following the manufacturer’s instructions. Ten parallel re-amplification reactions were required to obtain sufficient amounts of amplified DNA for hybridization. The samples were then purified using Qiaquick PCR purification columns (QIAGEN, Valencia, CA, USA), pooled and concentrated using a Millipore Centricon YM-10 (Millipore, Temecula, CA, USA). Concentrated samples were then re-suspended in 37 μl ddH_2_O and quantified using the ND-1000 (Nanodrop, Wilmington, DE, USA).

### DNA preparation, labeling and hybridization

In a 50 μl reaction, fragmentation of DNA was achieved by incubation of 30 μg genomic DNA with 0.0015 U RQ1 DNase I (Promega, Madison, WI, USA) per μg of DNA in the presence of: 0.175 mg/ml BSA, 10 mM Tris-acetate pH 7.5, 10 mM magnesium acetate, and 50 mM potassium acetate. Reactions were carried out for 30 minutes at 37°C followed 15 min at 99°C. Fragmentation profiles were examined by electrophoresis of 1.5 μl of fragmented product on a 2% agarose gel alongside a 50 bp DNA ladder (Fermentas, Glen Burnie, MD, USA) (Figure 3). Fragmented DNAs ranging between 25 to 250 bp were considered acceptable for hybridization.

The 3’ termini were end-labeled by addition of a biotinylated oligonucleotide (Affymetrix 7.5 mM Labeling Reagent) using terminal deoxynucleotidyl transferase (TdT) (Promega, Madison, WI, USA) as per a modified Affymetrix Labeling of Fragmented Double-Stranded DNA instructions [33]. The reaction was scaled up to accommodate 30 μg of fragmented DNA rather than the 7.5 μg fragmented cDNA. The BioPrime DNA Labeling System (Invitrogen, Carlsbad, CA, USA) was also tested for labeling DNA as per the manufacturer’s instructions.

Hybridizations were carried out following the protocol described in Affymetrix GeneChip® Whole Transcript Double-Stranded Target Assay (WTDSTA) Manual (pp. 51–52; Affymetrix 2005) with minor modification. Samples were comprised of 70 μl of fragmented and labeled DNA containing the elevated amount of DNA (30 μg total), the remaining components of the GeneChip® Hybridization Kit (Affymetrix, Santa Clara, CA, USA), adjusted to 1.1x standard volumes, and the 20x hybridization control from the Hybridization Control Kit (Affymetrix, Santa Clara, CA, USA) were added as per instructions for a total volume of 220 μl. After centrifugation, the microarray cassette was injected with 200 μl of the cocktail. Post hybridization, washing and staining were carried out as described in the Affymetrix GeneChip® WTDSTA Manual using program FS450_0001 (pp. 55–61 Affymetrix 2005). Hybridization intensities were measured using an Affymetrix GeneChip® Scanner 3000 with 7 G up-grade.

### Data processing and management

The raw .CEL files generated by the GCOS software (Affymetrix, Santa Clara, CA, USA) were subjected to background correction by using the Robust Multiarray Analysis (RMA) method and quantile normalization [34] of the Affymetrix package [35] with R and Bioconductor Affymetrix package [36]. A MySQL 5.0 database was developed to curate all the information regarding experiments, probe characteristics, hybridization data, and quality control data (https://pgfmars.ucdavis.edu/phpmyadmin). A web interface was designed using PHP scripts for retrieval of the data. All scripts of our “SPPscan Package for Lettuce GeneChip® Data Management, Processing and Analysis” and detailed descriptions can be obtained from http://chiplett.ucdavis.edu/.

### SPPdev detection algorithm

Perl scripts were developed for SPP detection. For hybridization of genomic DNA, the reference hybridization values in the SFPdev formula should be all of the lettuce probes on the microarray with the same GC content rather than those within a unigene as utilized by West *et al.*[5] for cDNA hybridizations. We therefore defined the SFPdev for each probe *i* of each unigene from each chip g as:

$$\text{SFPde}v_{i}^{g}=\frac{\left|Y_{i}-R\left(Y_{i}\right)\right|}{Y_{i}}\text{,}$$

Where *R*(*Y*_*i*_) is the average value of all the high quality probes on the same hybridized lettuce chip with the same GC content as probe *Y*_*i*_. The high quality probes were defined as those probes whose signal intensities exceeded those of a defined percentile of anti-genomic probes. The threshold value selected for our analysis was 90% (See Results).

The overlapping 2 bp tiling path on the lettuce chip allowed us to utilize the average weighted signal from multiple probes covering individual positions in each unigene. Deviation in hybridization intensities was calculated using a 2 bp sliding window incorporating data from all high quality probes covering each position. For each 2 bp position, *s*, in a unigene covered by at least one probe*,* we calculated the single position polymorphism deviation (SPPdev) as:

$$\text{SFPde}v_{s}=\underset{}{\Sigma }w_{k}\text{SFPde}v_{k}\text{,}$$

Where *w*_*k*_ is the weighting factor for the high quality probe *k* that covers position *s* in the unigene. The weighting factor takes into account the distance from the central nucleotide of each probe to the evaluated position (Figure 5). The weighting factor was determined empirically by analyzing the hybridization behavior of probes covering a training set of 1,000 SNPs obtained from the alignment of multiple ESTs from *L. sativa* cv. Salinas with *L. serriola* acc. US96UC23 (See Results). All scripts are available from http://chiplett.ucdavis.edu/.

### Statistical evaluation of putative SPPs

For each evaluated position of a unigene, SPPdev values were obtained for each replicated microarray of the two genotypes (*L. sativa* cv. Salinas and *L. serriola* acc. US96UC23). To detect putative SPPs within each unigene, we used the test statistic, *R*_*s*_ defined as the SPPdev ratio for position *s*:

$$R_{s}=\frac{\overset{n}{\underset{j=1}{\Sigma }}\text{SFPde}v_{\text{sj}}^{\text{SAL}}/n}{\overset{n}{\underset{j=1}{\Sigma }}\text{SFPde}v_{\text{sj}}^{\text{SER}}/n}$$

Where *n* is the number of replicated microarray chips for each genotype. If the value of *R*_*s*_ was much larger or smaller than what was expected by chance, then the position *s* of the unigene under study was predicted to have a SPP. A p-value was then assigned to each *R*_*s*_ according to the null distribution of *R*_*s*_ which was estimated by permutation [37]. The follo-wing inference procedure was carried out for each unigene:

1) Arrange rows for all the probes of the unigene and columns for all the replicates of the two genotypes.

2) For the *b*^th^ permutation, b = 1, 2,…B:

a) Permute the columns of the data matrix obtained in S1

b) Compute $R_{s}^{b}$ for each position *s*, *s =* 1, 2,…m.

3) After carrying out the B permutations, sort all the $R_{s}^{b}$ values obtained from Step 2 in increasing order. The permutation p-value for testing the null hypothesis (that no polymorphism exists at position *s*) is determined by:

the background-corrected and quantile-normalized hybridization data in a matrix with

4) $p_{s}=\{\begin{array}{c} \frac{\left\{R_{s}^{b}>R_{s}\text{ and }R_{s}^{b}<1/R_{s}:s=1,2,\ldots m;\text{b}=1\text{,2,}\ldots \text{B}\right\}}{B*m}\text{if }R_{s}>1\\ \frac{\left\{R_{s}^{b}<R_{s}\text{ and }R_{s}^{b}>1/R_{s}:s=1,2,\ldots m;\text{ b}=1\text{,2,}\ldots \text{B}\right\}}{B*m}\text{if }R_{s}<1 \end{array}$< 0.01.

5) Obtain a FDR-adjusted p-value [38], P^*^_s_ for each ps.

6) A position s is claimed to be a putative SPP if P^*^_s_ < 0.01.

Note that the null distribution of *R*_*s*_ for each position *s* is assumed to be the same. Therefore, the null distribution of *R*_*s*_ is estimated by pooling the permutation distributions of *R*_*s*_ at all positions.

### Alternative SPP detection algorithm (Modified SFP Algorithm; MSA)

An algorithm to identify SFPs in complex genomes was described by Borevitz *et al.*[1] and implemented in R (http://www.r-project.org). This algorithm was modified to interrogate every 2 bp position on the lettuce GeneChip® instead of individual probes. The MSA algorithm was implemented in Perl because R could not handle our large data set and to facilitate parallelization of analysis on a Linux computer cluster. In MSA, we calculated the *D-stat* statistics at every 2 bp position as in Equation 1, instead of calculation of the *D-stat* statistics for each probe as in the original algorithm. The FDR calculation is described in Borevitz *et al*. (2003; http://naturalvariation.org/methods/); however, quantile function of R which is used in FDR calculation was implemented in Perl. The MSA Perl scripts can be accessed through (http://chiplett.ucdavis.edu).

(1) $$D-stat=\frac{\text{Mean}\;\text{of}\;\text{Genotype}\;A-\text{Mean}\;\text{of}\;\text{Genotype}\;B\;}{S_{i}\;\text{this}\;\text{position}-S_{0}}$$

Where:

Mean of parent A or B at a given 2 bp position is natural logarithm (L_*n*_) of weighted average intensity of all probes that interrogating a position,

*S*_*i*_ is calculated for each position but a* constant is a constant value for all positions,

$$\begin{array}{c} S_{i}=\sqrt{a^{*}\text{Constant}\times (\text{Sum of Squares of Genotype A}+\text{Sum of Squares of Genotype B})}\\ a^{*}\text{constant}=\frac{\frac{1}{\text{No. of replications Genotype A}}+\frac{1}{\text{No. of replications Genotype B}}}{(\text{No. of replications Genotype A }+\text{No. of Replications Genotype B})-2} \end{array}$$

*S*_*0*_ is a constant for all positions, which is the median of all *S*_*i*_ values for the entire 2 bp positions on the chip (>10,000,000 positions).

### Transcriptome sequencing

RNA was isolated from multiple tissues and treatment conditions (http://cgpdb.ucdavis.edu/cgpdb2/data_info_files/CGP_Library_Construction_V02.html) and pooled in equimolar concentration for each of the two genotypes. cDNA was synthesized and prepared for sequencing following protocols as described [39]. *L. sativa* cv. Salinas and *L. serriola* acc. US96UC23 were sheared and selected for fragments around 260 bp and 200 bp respectively. Fragments were normalized using duplex specific nuclease (DSN, Evrogen, Moscow, Russia) after denaturation and re-association for five hours to reduce high copy sequences digesting double stranded DNA. The *L. sativa* cv. Salinas library was sequenced in one direction while the *L. serriola* acc. US96UC23 library was sequenced as paired-ends. Sequences are available in the Sequence Read Archive at NCBI (Study numbers SRP004854 and SRP008310).

### Validation

Using BWA [40] and SAMtools [41] to identify a set of high quality SNPs, heterozygous positions, and InDels in the IGA set. Custom scripts were used to identify false positive SPPs identified by each of the SFPdev and MSA methods by comparison to the IGA set, the SNPs mined from the EST assembly used to develop the array, and SPP markers mapped in the core mapping population [20]. A SPP was considered a true positive if SNPs were within 8 bp of the SPP ranges. An 8 bp range on either side of detected SPPs was chosen to account for detection of SPPs with the overlapping 25 bp oligo design and empirically determined sensitivity of oligos (Figure 5). Additionally, if the SPP range in the pair-wise comparison overlapped the SPP range mapped within the core RIL population, it was considered real. SPPs with no IGA sequence were excluded as we were not able to identify if they were true or false SPPs.

In order to identify if the apparent SPP was present in multiple contigs, each SPP plus and minus 8 bases on either side of the SPP range was compared using BLAST to three separate sets of sequences: a lettuce whole genome assembly of shotgun Illumina reads (Michelmore *et al.,* unpublished data), a gene space assembly (Kozik, unpublished data) and the EST assembly used for the array design (http://compgenomics.ucdavis.edu) using BLASTn [42]. To maintain confidence in the BLAST hits there had to be a minimum of 95% of the subject in the query with no more than two mismatches. SPPs identified as duplicated loci were removed from the analysis. SPPs detected in the SFPdev pair-wise comparison method that had a SFPdev ratio greater than one were removed as we empirically determined a 79% false positive rate through our validation procedures.

### Diversity panel analysis

As described in Truco *et al.*[20] the RIL algorithm calculates the SPPdev value for each array and plots that value for each position. The distribution of data points for each position is then evaluated for a bimodal distribution while maximizing the number of individual arrays under each peak. Those that fall between the identified populations of the bimodal distribution, or are completely missing, were treated as a missing data point (−). Arrays with hybridization signals in the higher part of the distribution were designated the A allele while those with lower hybridization values (because they deviated from the probe sequence), were designated the B allele. Data points for each array were then summarized based for each individual or cultivar as follows. Marker calls based on three replicates were designated as A, B, C, D, I (inconsistent), or – (missing). An A marker call resulted from an A in all three replicates. A B marker call resulted from a B in all three replicates. C calls were not A and were missing in one of the three reps i.e. (B/B/-) and D was not B and missing one of the three reps i.e. (A/A/-). An I was any combination of the three chips that contained two missing and an A or B or, any combination that included an A and B call from one of the three reps. – was missing data from all three reps.

### Phylogenetic analysis

Utilizing the package PHYLIP-3.69 [21] the SPP markers were treated as restriction site markers and scored as 1 or 0. The SeqBoot module was used to create 100 re-samples data sets for bootstrap calculation. The resulting 100 replicates were used to create distance matrices subsequently used for construction of phylogenetic trees with the Fitch module. Global rearrangement and randomized input order of species with 10 jumbles for each of the multiple data sets was used to construct 100 trees for use in building a consensus tree with bootstrap values. The Consense module was then used with species *L. perennis* used as an out-group root. The resulting consensus tree was then visualized with Mega4 [43] and branches were rotated for legibility. See Additional file 9: Table S2.

### Principal component analysis

For principal component analysis, markers within contigs (9,513) that were common to the *L. sativa* cv. Salinas by *L. serriola* acc. US96UC23 map [20] were converted to haplotype frequencies using custom Perl scripts. Frequencies were calculated as the number of times the haplotype occurs in the panel for a given contig divided by the total number of genotypes in the panel. A limitation on the number of markers to use with the SAS/STAT software required us to reduce our set of markers to 10,000 markers or less. Creating haplotypes for all markers within a contig allowed us to reduce the number of input data points to 8,381 while retaining more genetic information than a randomly selected marker for each mapped contig. The markers used for calculation of haplotype frequencies in the entire DP were then filtered to include those polymorphic within *L. sativa* lines only and haplotype frequencies were calculated for 3,311 contigs to resolve *L. sativa* varieties. Frequencies of haplotypes in the DP panel were used as input for principal component analysis using the SAS/STAT® software’s PRINCOMP procedure (Version 9.1.3, SAS Institute, Cary, NC). Eigenvalues for each of the first three principal components were calculated for each genotype and an analysis of variance (ANOVA) and means separation using Students t-test were performed using JMP (JMP, Version 7. SAS Institute Inc., Cary, NC, 1989–2007) to determine if species and/or classes were significantly different. The two data sets described above were used to differentiate lettuce species and lines. See Additional file 10: Table S3 and Additional file 11: Table S4.

## Competing interests

The authors declare that they have no competing interests.

## Authors’ contributions

KS optimized DNA extraction protocols, labeling methods, and processed all samples and lead writing of the manuscript. HL performed initial hybridization analysis and algorithm development, and helped draft the manuscript. AK participated in array design and study conception. DC aided with protocol development, array design and study conception. HA contributed to algorithm development and bioinformatics analysis of data. XC aided in statistical evaluation. XT participated in protocol development. TH participated in protocol development. SRC aided in algorithm development and performed validation procedures. MJT aided in diversity panel development and study conception. RM conceived the study and participated in design, data interpretation and coordination. AVD conceived the study and participated in design, data interpretation and coordination. All authors read and approved the final manuscript.

## Supplementary Material

Additional file 1 **Figure S1.** A representation of the tiling path across a contig shows probes constructed for both sense and anti-sense strands at a 4 bp stagger deviated by 2 bp to result in final 2 bp stagger. Click here for file

Additional file 2 **Figure S2.** A histogram showing the number of probes per unigene. Click here for file

Additional file 3 **Figure S3.** A histogram showing the frequency of probes separated by GC bin from five to nineteen guanines or cytosines. Click here for file

Additional file 4 **Figure S4.** Pair-wise scatter plots of 600,000 random RMA background corrected hybridization values comparing WGA amplified DNase I fragmented, end-labeled genomic DNA (SAL_WGA) versus untreated DNase I fragmented, endlabeled genomic DNA (SAL_30_01, SAL_30_02, SAL_30_New_1, SAL_30_New_2). Coefficient of determination (R2) values opposite their scatter plots indicates a bias in treatments. Click here for file

Additional file 5 **Figure S5.** Hybridization values per GC bin of BioPrime labeled/amplified samples, two reactions combined (Bioprime_2x), WGA amplified, dUTP incorporated, APE I UDG fragmented end-labeled DNA and DNase I end labeled DNA are compared to show the effect of techniques on hybridization intensities. BioPrime labeled/amplified samples show an increased in hybridization values in probes with higher GC content compared to other methods. Click here for file

Additional file 6 **Figure S6.** SPPdev values are plotted along a contig. The orange lines compared to the black lines show the effect on SPP calls when probes hybridizing below the 90th percentile of anti-genomic are removed. Click here for file

Additional file 7 **Figure S7.** Enlarged view of *L. sativa* clade from Tree 2 phylogram. Branch lengths represent relative genetic distance. Click here for file

Additional file 8 **Table S1.** An analysis of variance and least squares mean separations were performed using JMP on genotypes, separating species or classes within *L. sativa* when possible. Least squares means separation using Students t-test separated classes and species showing significant differences between species/classes with different letters. Left column: The three tables show the analysis of variance for all genotypes in the DP separated by species. Moving downward in the column shows the ANOVA for principle components one through three respectively. Each of the first three principle components were significant at <0.001. Right Column: The three tables show the ANOVA for each of the first three principle components separated by horticultural type. Only principle components one and three are significantly different in this analysis. Click here for file

Additional file 9 **Table S2.** A table of SPP data for each of the markers used in the creation of phylogenetic trees. Click here for file

Additional file 10 **Table S3.** A table of haplotype frequencies used for the principle component analysis of all individuals in the diversity panel. Click here for file

Additional file 11 **Table S4.** A table of haplotype frequencies used for the principle component analysis of *L. sativa*. Click here for file
