# Supplementary material for: Development and application of a 6.5 million feature Affymetrix Genechip® for massively parallel discovery of single position polymorphisms in lettuce (Lactuca spp.)
Source: BMC Genomics. 2012 May 14;13:185. doi: 10.1186/1471-2164-13-185 (PMC3490809; doi:10.1186/1471-2164-13-185)
Supplement: Additional file 1 — Figure S1. A representation of the tiling path across a contig shows probes constructed for both sense and anti-sense strands at a 4 bp stagger deviated by 2 bp to result in final 2 bp stagger. [file 1471-2164-13-185-S1.pdf]

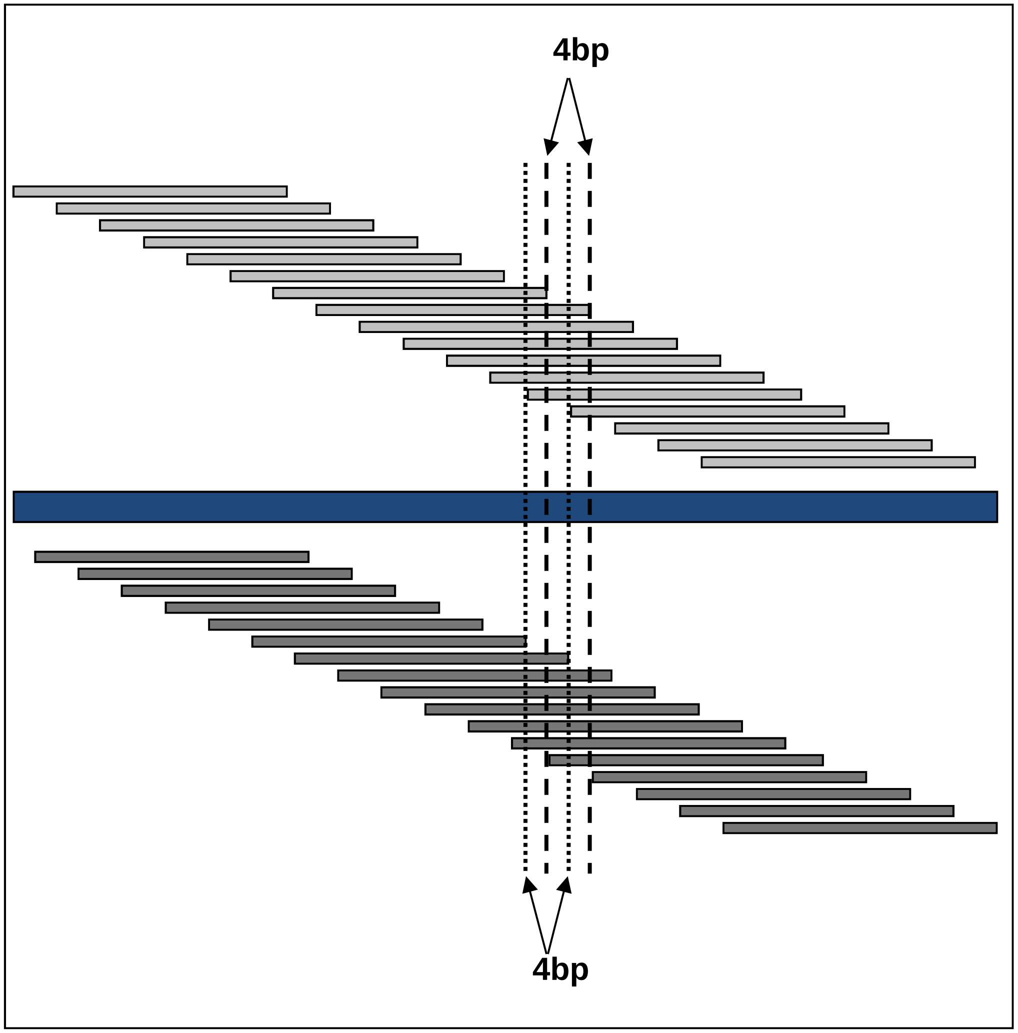

Figure S1 A representation of the tiling path across a contig shows probes constructed for both sense and anti-sense strands at a 4 bp stagger deviated by 2 bp to result in final 2 bp stagger.
