# Supplementary figures and images for: Development and application of a 6.5 million feature Affymetrix Genechip® for massively parallel discovery of single position polymorphisms in lettuce (Lactuca spp.)
Source: BMC Genomics. 2012 May 14;13:185. doi: 10.1186/1471-2164-13-185 (PMC3490809; doi:10.1186/1471-2164-13-185)

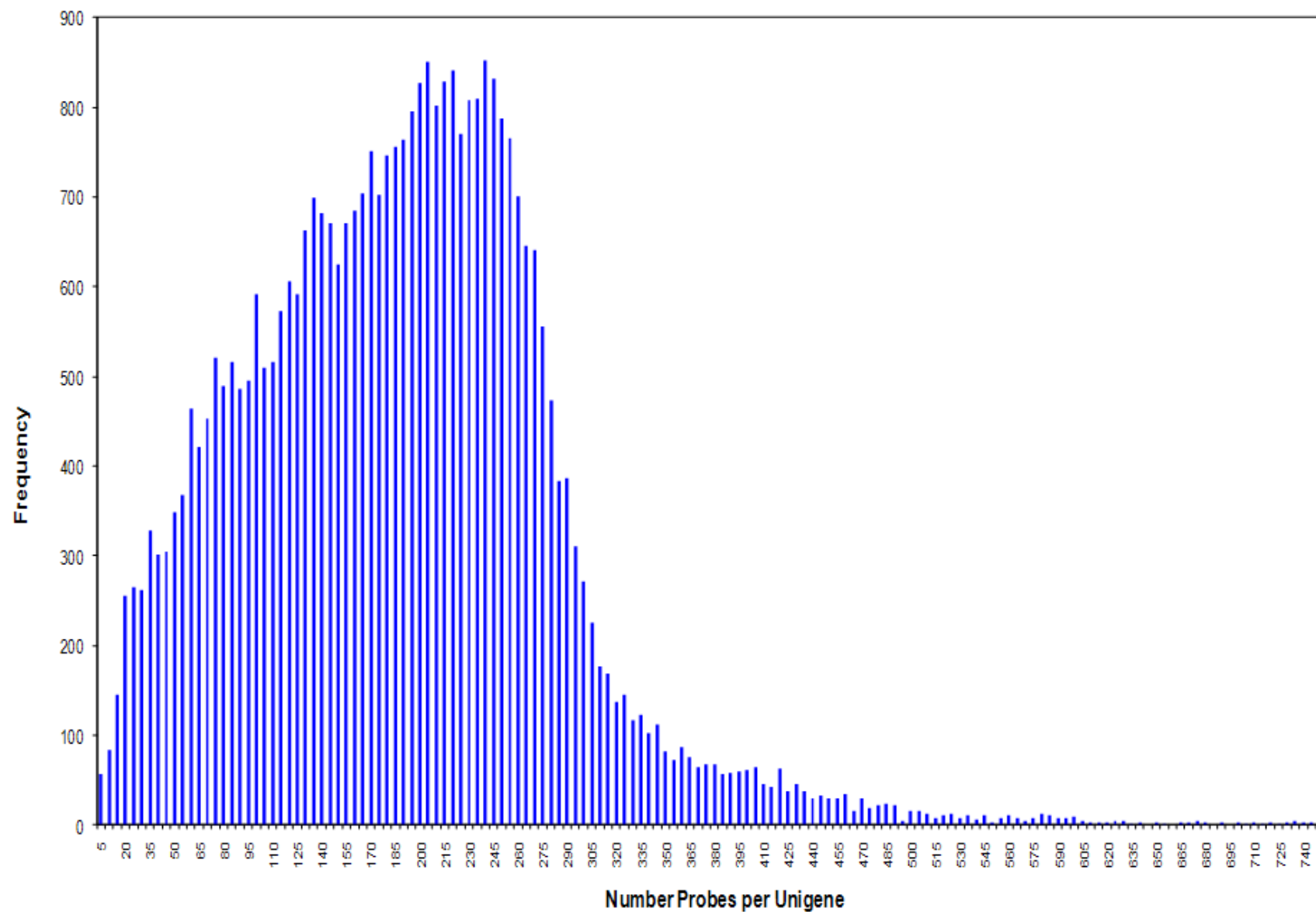

**Figure S2** A histogram showing the number of probes per unigene.

Supplement: Additional file 2 — Figure S2. A histogram showing the number of probes per unigene. [file 1471-2164-13-185-S2.pdf]
