# Supplementary material for: Development and application of a 6.5 million feature Affymetrix Genechip® for massively parallel discovery of single position polymorphisms in lettuce (Lactuca spp.)
Source: BMC Genomics. 2012 May 14;13:185. doi: 10.1186/1471-2164-13-185 (PMC3490809; doi:10.1186/1471-2164-13-185)
Supplement: Additional file 3 — Figure S3. A histogram showing the frequency of probes separated by GC bin from five to nineteen guanines or cytosines. [file 1471-2164-13-185-S3.pdf]

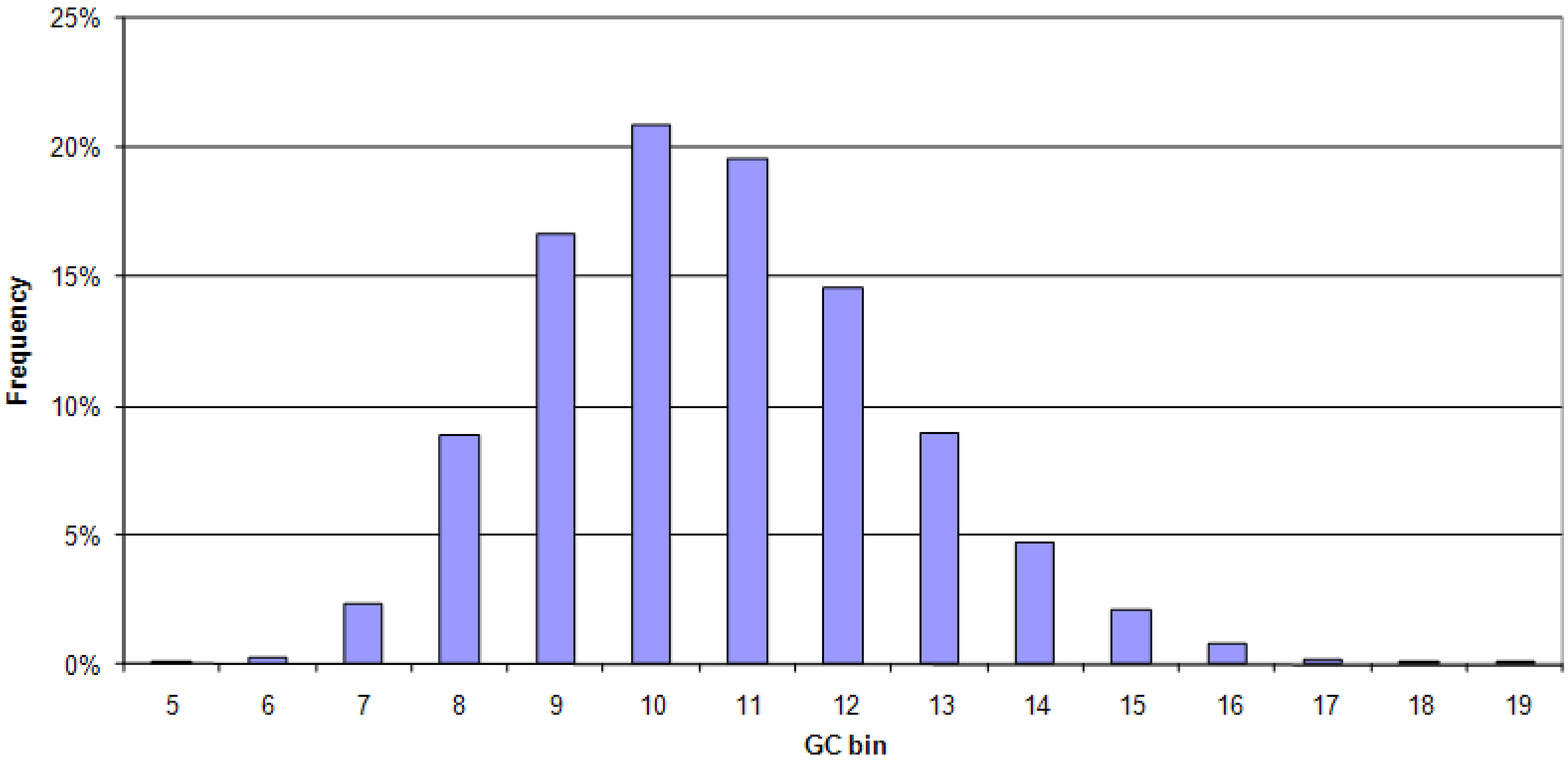

Figure S3 A histogram showing the frequency of probes separated by GC bin from five to nineteen guanines or cytosines.
