# Supplementary material for: Development and application of a 6.5 million feature Affymetrix Genechip® for massively parallel discovery of single position polymorphisms in lettuce (Lactuca spp.)
Source: BMC Genomics. 2012 May 14;13:185. doi: 10.1186/1471-2164-13-185 (PMC3490809; doi:10.1186/1471-2164-13-185)
Supplement: Additional file 4 — Figure S4. Pair-wise scatter plots of 600,000 random RMA background corrected hybridization values comparing WGA amplified DNase I fragmented, end-labeled genomic DNA (SAL_WGA) versus untreated DNase I fragmented, endlabeled genomic DNA (SAL_30_01, SAL_30_02, SAL_30_New_1, SAL_30_New_2). Coefficient of determination (R2) values opposite their scatter plots indicates a bias in treatments. [file 1471-2164-13-185-S4.pdf]

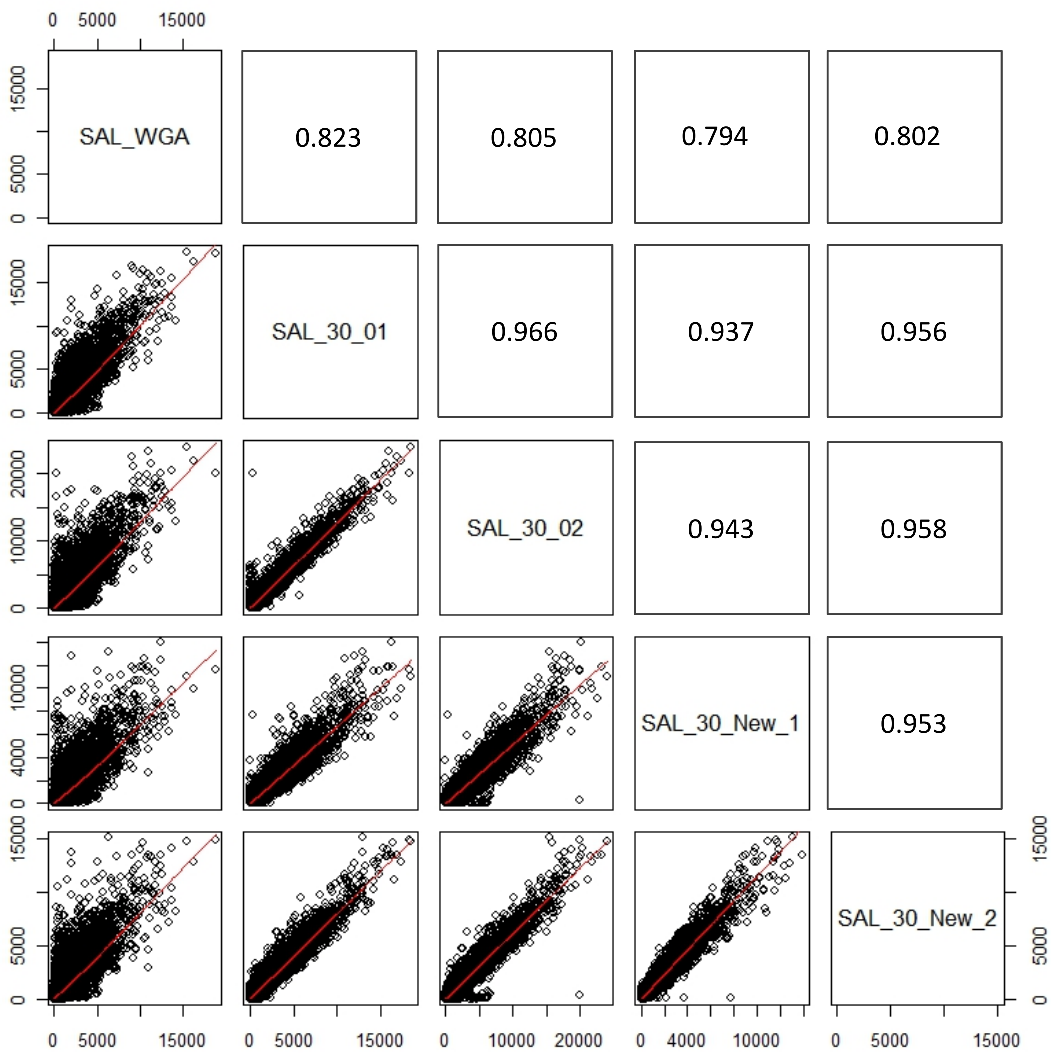

Figure S4 Pair-wise scatter plots of 600,000 random RMA background corrected hybridization values comparing WGA amplified DNase I fragmented, end-labeled genomic DNA (SAL_WGA) versus untreated DNase I fragmented, end-labeled genomic DNA (SAL_30_01, SAL_30_02, SAL_30_New_1, SAL_30_New_2). Coefficient of determination (R^2) values opposite their scatter plots indicates a bias in treatments.
