# Supplementary material for: Development and application of a 6.5 million feature Affymetrix Genechip® for massively parallel discovery of single position polymorphisms in lettuce (Lactuca spp.)
Source: BMC Genomics. 2012 May 14;13:185. doi: 10.1186/1471-2164-13-185 (PMC3490809; doi:10.1186/1471-2164-13-185)
Supplement: Additional file 5 — Figure S5. Hybridization values per GC bin of BioPrime labeled/amplified samples, two reactions combined (Bioprime_2x), WGA amplified, dUTP incorporated, APE I UDG fragmented end-labeled DNA and DNase I end labeled DNA are compared to show the effect of techniques on hybridization intensities. BioPrime labeled/amplified samples show an increased in hybridization values in probes with higher GC content compared to other methods. [file 1471-2164-13-185-S5.pdf]

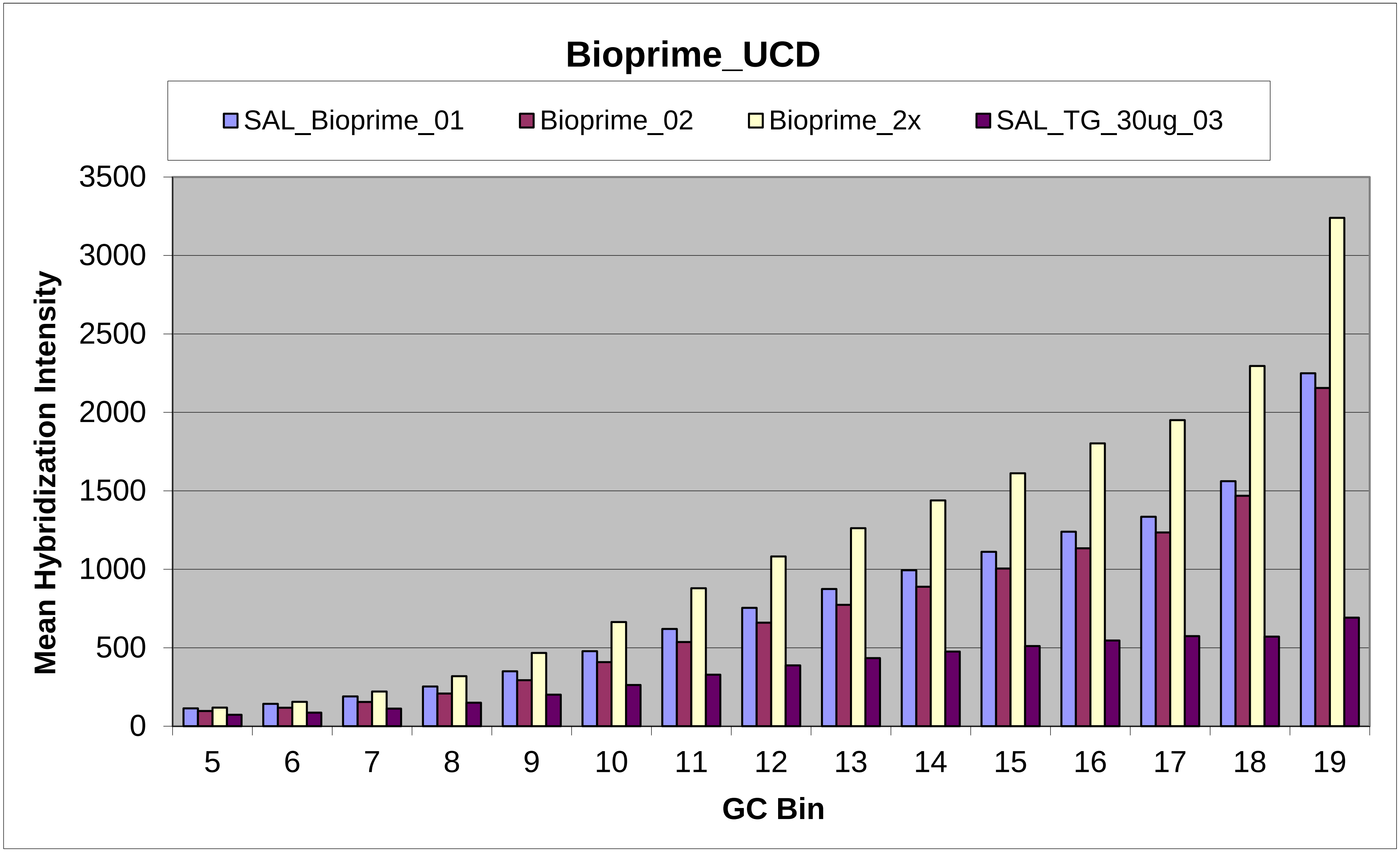

Figure S5 Hybridization values per GC bin of BioPrime labeled/amplified samples, two reactions combined (Bioprime_2x), WGA amplified, dUTP incorporated, APE I UDG fragmented end-labeled DNA and DNase I end labeled DNA are compared to show the effect of techniques on hybridization intensities. BioPrime labeled/amplified samples show an increased in hybridization values in probes with higher GC content compared to other methods.
