# Supplementary material for: Development and application of a 6.5 million feature Affymetrix Genechip® for massively parallel discovery of single position polymorphisms in lettuce (Lactuca spp.)
Source: BMC Genomics. 2012 May 14;13:185. doi: 10.1186/1471-2164-13-185 (PMC3490809; doi:10.1186/1471-2164-13-185)
Supplement: Additional file 6 — Figure S6. SPPdev values are plotted along a contig. The orange lines compared to the black lines show the effect on SPP calls when probes hybridizing below the 90th percentile of anti-genomic are removed. [file 1471-2164-13-185-S6.pdf]

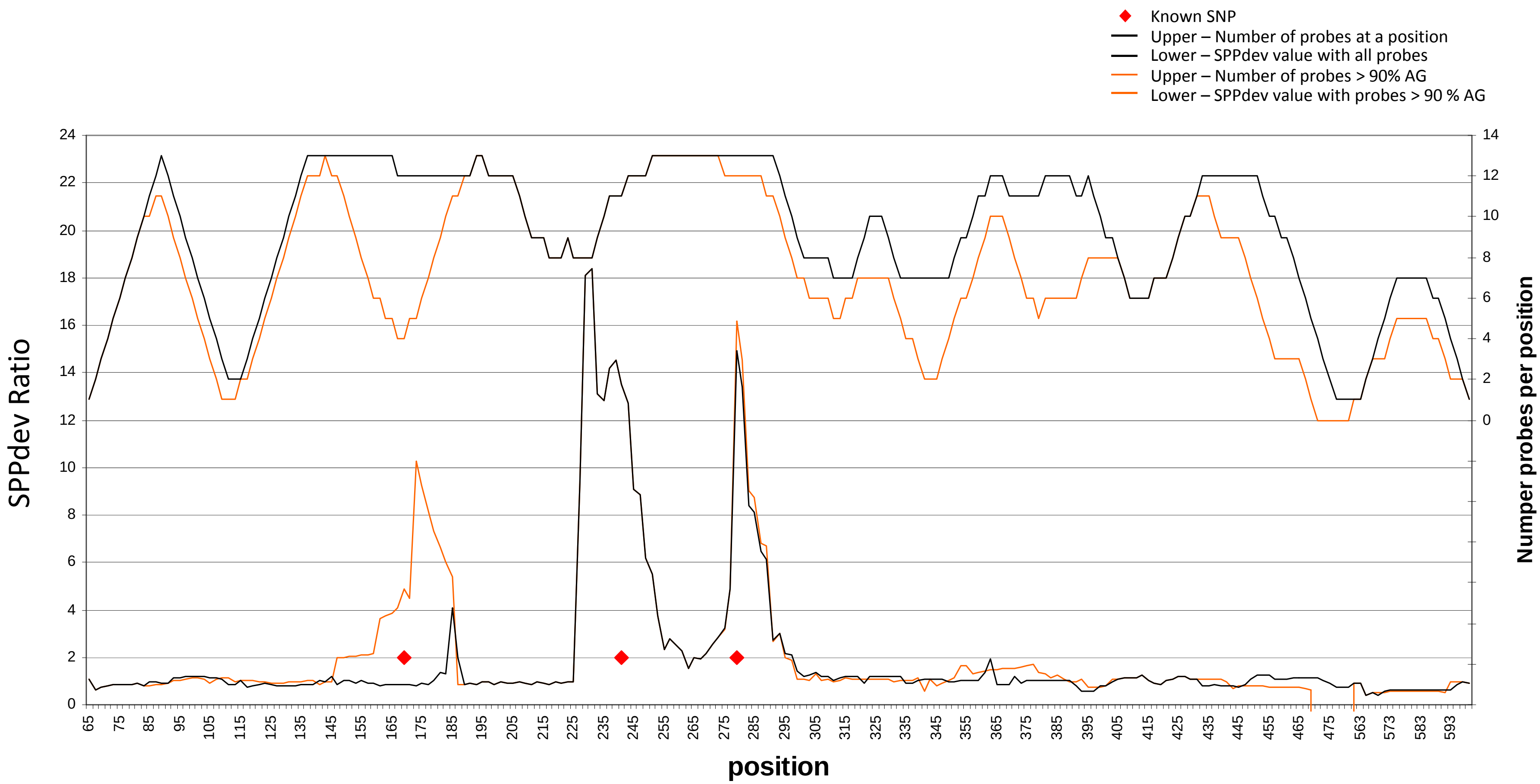

Figure S6 SPPdev values are plotted along a contig. The orange lines compared to the black lines show the effect on SPP calls when probes hybridizing below the 90th percentile of anti-genomic are removed.
