# Supplementary material for: Development and application of a 6.5 million feature Affymetrix Genechip® for massively parallel discovery of single position polymorphisms in lettuce (Lactuca spp.)
Source: BMC Genomics. 2012 May 14;13:185. doi: 10.1186/1471-2164-13-185 (PMC3490809; doi:10.1186/1471-2164-13-185)
Supplement: Additional file 7 — Figure S7. Enlarged view of L. sativa clade from Tree 2 phylogram. Branch lengths represent relative genetic distance. [file 1471-2164-13-185-S7.pdf]

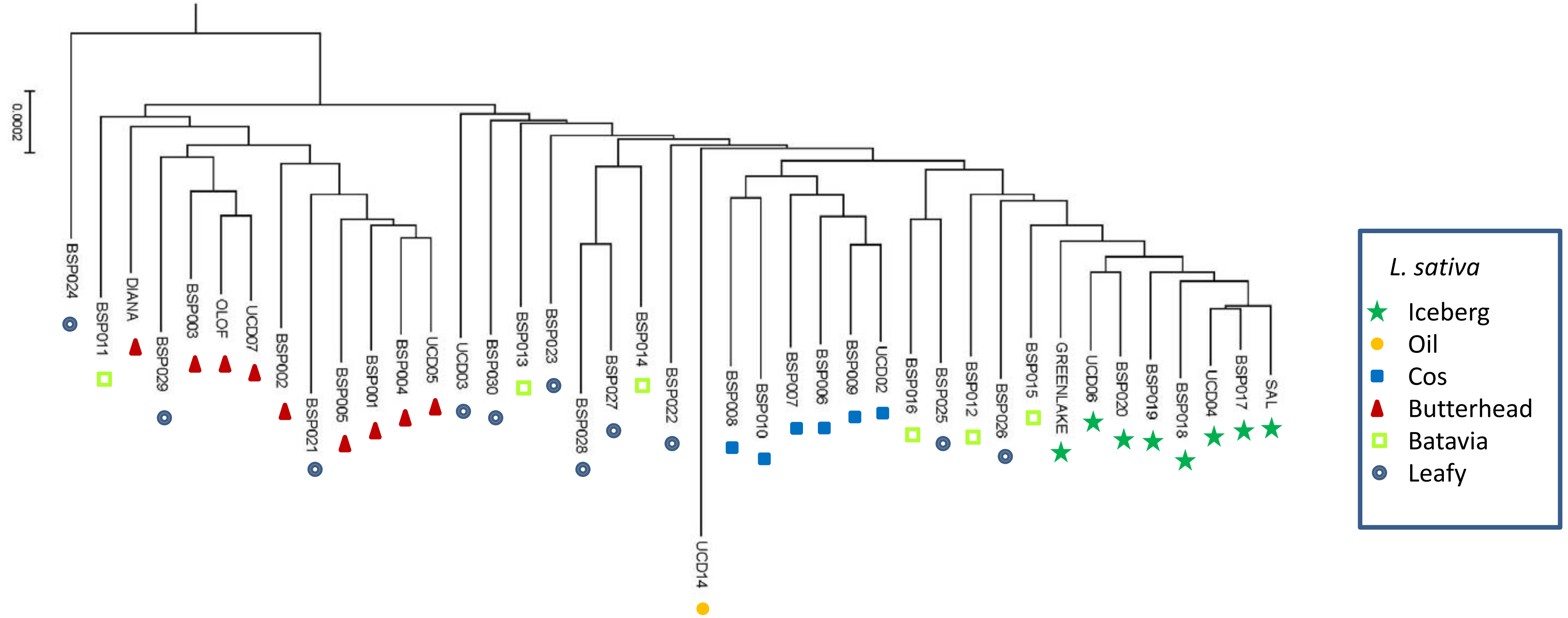

Figure S7 Enlarged view of *L. sativa* clade from Tree 2 phylogram. Branch lengths represent relative genetic distance.
